# Competitive Outcome of *Daphnia-Simocephalus* Experimental Microcosms: Salinity versus Priority Effects

**DOI:** 10.1371/journal.pone.0070572

**Published:** 2013-08-05

**Authors:** Cláudia Loureiro, Joana L. Pereira, M. Arminda Pedrosa, Fernando Gonçalves, Bruno B. Castro

**Affiliations:** 1 Departamento de Biologia & CESAM, Universidade de Aveiro, Aveiro, Portugal; 2 Unidade de I&D n° 70/94– Química-Física Molecular/FC, Departamento de Química, Faculdade de Ciências e Tecnologia da Universidade de Coimbra, Coimbra, Portugal; Texas Tech University, United States of America

## Abstract

Competition is a major driving force in freshwaters, especially given the cyclic nature and dynamics of pelagic food webs. Competition is especially important in the initial species assortment during colonization and re-colonization events, which depends strongly on the environmental context. Subtle changes, such as saline intrusion, may disrupt competitive relationships and, thus, influence community composition. Bearing this in mind, our objective was to assess whether low salinity levels (using NaCl as a proxy) alter the competitive outcome (measured as the rate of population biomass increase) of *Daphnia-Simocephalus* experimental microcosms, taking into account interactions with priority effects (sequential species arrival order). With this approach, we aimed to experimentally demonstrate a putative mechanism of differential species sorting in brackish environments or in freshwaters facing secondary salinization. Experiments considered three salinity levels, regarding NaCl added (0.00, 0.75 and 1.50 g L^−1^), crossed with three competition scenarios (no priority, priority of *Daphnia* over *Simocephalus*, and vice-versa). At lower NaCl concentrations (0.00 and 0.75 g L^−1^), *Daphnia* was a significantly superior competitor, irrespective of the species inoculation order, suggesting negligible priority effects. However, the strong decrease in *Daphnia* population growth at 1.50 g L^−1^ alleviated the competitive pressure on *Simocephalus*, causing an inversion of the competitive outcome in favour of *Simocephalus*. The intensity of this inversion depended on the competition scenario. This salinity-mediated disruption of the competitive outcome demonstrates that subtle environmental changes produce indirect effects in key ecological mechanisms, thus altering community composition, which may lead to serious implications in terms of ecosystem functioning (e.g. lake regime shifts due to reduced grazing) and biodiversity.

## Introduction

Competition is a major driving force in freshwater systems, especially given the cyclic nature and dynamics of planktonic food webs [1], [2]. While intra-specific competition is important in defining equilibrium in population dynamics, inter-specific competition tends to be destabilizing, causing ecological exclusion of one or the other competitor(s) [3], [4]. Inter-specific competition generally translates into the mutual inhibition of growth rate among populations of different species that have common requirements for shared and limiting resources. Competition between populations of freshwater cladocerans can be responsible for shifts in competitor’s life-history, in terms of density, growth, juvenile survival and clutch-size [5], leading to a co-existence scenario with different demographic cycles [5], [6].

Regulation of cladoceran community structure is modulated by colonization and re-colonization events from the ephippial egg bank [7], [8]. Competition is especially important in the initial species assortment [2], [9], [10], which depends strongly on the initial species and gene pool (producing so-called founder effects [7], [11]), as well as the environmental conditions of the system. Under such scenarios, the order at which species appear in the system may configure priority effects, in which species that appear first have a competitive advantage over latecomers [8], [12]. Priority effects are defined as the impact that a particular species can have on community development due to prior arrival (or hatching) at a site, and they usually result from resource and niche monopolization of early colonizers [8], [13].

The environmental context is known to impact the strength of priority effects or even superimpose them (e.g. [8]). Previous experiments with *Daphnia* [14], [15] have shown that the environmental context influences the competitive outcome. Louette and De Meester [8] showed that predation may alter the competitive outcome of inter-specific relationships. Using plants as experimental subjects, several authors have shown that competitive ability or intensity is alleviated under environmental stress (e.g. [16]). Also, Emery et al. [17] demonstrated that stress tolerators were consistently dominant competitors under some circumstances. The reasonable conclusion is that environmental stress, either abiogenic or biogenic, may alter radically the expected outcome of species sorting, a key process in the population dynamics of freshwater cladoceran populations.

Salinity is an abiotic environmental stressor that can radically alter freshwater community structure (e.g. [18], [19]). In zooplankton, such community changes can occur at low salinity levels [20], [21], [22], [23]. Salinization of freshwaters, which is a predicted consequence of global climate change and groundwater overexploitation [24], represents serious implications for ecosystem functioning. For example, lake regime shifts from clear to turbid water may occur in brackish lakes [25] due to removal of large herbivores (either eliminated directly by salinity or via altered fish community composition – see [25]). At lethal salinity levels (>2), sensitive species are purely eliminated or are unable to hatch. At lower levels, however, salinity could disrupt competitive relationships, with brackish conditions favouring different species composition than in freshwater conditions. So far, there is no experimental evidence for this in the literature.

Bearing this in mind, our objective was to assess whether low salinity levels (using NaCl as a proxy) alter the competitive outcome of a *Daphnia-Simocephalus* experimental system, taking into account interactions with priority effects (sequential species arrival order). It is expected that salinity alters the competitive outcome of inter-specific relationships, provided that there are slight differences in halotolerance between competitor species; however, it is hypothesized that priority effects (inoculation order of the competitor species) may confer some protection to the less halotolerant species. With this approach, we aim to experimentally confirm the hypothesized mechanism of differential species sorting in brackish environments or in freshwaters facing secondary salinization.

## Materials and Methods

### Cultures and Test Organisms

Monoclonal cultures of *Simocephalus vetulus* (Müller, 1776) and *Daphnia galeata* Sars, 1864 were reared in the laboratory for several generations (more than one year). They were both collected from freshwater reservoirs for previous experiments (clone LM64 [26] and clone B, respectively), using a plankton net. Both reservoirs were characterised for being eutrophic, bearing no previous records of above-zero salinity, and populated with planktivorous and omnivorous fish. These cladocerans are common and ubiquitous species in temperate lakes and reservoirs. No permits were necessary for collecting living plankton in the sampled reservoirs (Lagoa de Mira and Albufeira de Belver), as these organisms are not under specific conservation regulations and the land is public domain. No endangered or protected species were disturbed or involved in the present study.

Cladoceran cultures were reared in moderately hard reconstituted water (123 mg L^−1^ MgSO_4_·7H_2_O, 96 mg L^−1^ of NaHCO_3_, 60 mg L^−1^ CaSO_4_·2H_2_O, e 4 mg L^−1^ KCl, *sensu* ASTM [27] and USEPA [28]), supplemented with 4 mL L^−1^ of a standard organic additive (algal extract) and vitamins (for further details, see [26], [29]). Reconstituted water was prepared with UV-sterile deionised water (conductivity <10 µS cm^−1^) obtained with mixed-bed ion exchange resins, after pre-filtration through particle filters and activated carbon cartridges. Cultures were maintained under a temperature of 20±2°C and a 16h^L^:8h^D^ photoperiod, and organisms were fed three times a week (Monday, Wednesday, Friday) with a *Pseudokirchneriella subcapitata* ration of 1.5×10^5^ cells mL^−1^ (for more information on algal culture and ration, see [26], [30], [31]).

### Competition Experiment

The experiment was performed in transparent plastic buckets (experimental microcosms; internal diameter: 16–19 cm; height: 21 cm) containing 4 L of test solution. Three salinity levels were used (0.00, 0.75, and 1.50 g of NaCl per L), by dissolving reagent-grade NaCl (salinity proxy) in the culture medium. In order to simulate three different competition scenarios, we manipulated the order of inoculation of competitor species (priority effects). In two of the treatments, one species was introduced at day 0 and the other at day 10, thus simulating priority of *D. galeata* (D) over *S. vetulus* (S) (treatment D>S) and vice-versa (S>D); in the third treatment (S|D), both species were introduced at day 0 (i.e. no priority). Competition scenario (3 levels) was fully crossed with NaCl concentration (3 levels) in a total of 9 experimental treatments. Each experimental treatment was replicated 3 times, bearing a total of 27 experimental units (microcosms). Experiments were initiated with young (5–6 d old) females, to allow proper manipulation and visualization. All microcosms were inoculated with 10 individuals of each species, following the chronological order above.

A semi-static approach was used, by renewing 50% of test medium every week; this was done by siphoning 1 L of water from each microcosm, assuring no organisms were removed, and adding 1 L of fresh medium, two times a week (Mondays and Thursdays). After medium renewal, organisms were fed with a *P. subcapitata* ration of 0.75×10^5^ cells mL^−1^. This represents a less concentrated and a less frequent algal ration than in cultures, so that food becomes somewhat more limiting towards the final stage of the competition experiment, when both competitor populations are established. All experiments were carried out under a temperature of 20±2°C and a 16h^L^:8h^D^ photoperiod. Once a week, UV-sterile deionised water was added to compensate for evaporation losses, and conductivity, pH and oxygen were measured for quality assurance criteria. All treatments were terminated at day 30, regardless of the day the animals were inoculated in the microcosms.

At the end of the experiment (day 30), abundance and biomass of *Daphnia* and *Simocephalus* populations were estimated. Each microcosm was filtered through a 55-µm-mesh plankton net and the corresponding residue was preserved in 96% ethanol. All organisms were sorted into species and size classes (large adults, ≥1.8 mm; 1.2 mm≤small adults<1.8 mm; juveniles, <1.2 mm), and then counted under a stereoscope. Body length measurements (from top of head to base of caudal spine) were taken, using a stratified approach: all large adults were measured, while lengths of small adults and juveniles were measured in sub-samples of 50 individuals. Biomass estimates for both *Daphnia* and *Simocephalus* were obtained from a general length-weight relationship for daphniids (as recommended by [32]):
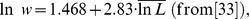
where ln *w* is the natural logarithm of dry weight (in µg) and 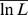 is the geometric mean length (mm) of individuals in the sample [34]. Mean individual weight was calculated for each stratum (large adults, small adults, juveniles), and total biomass (in µg L^−1^) was estimated taking into account the counts for each size class.

The rate of population biomass increase (r_b_, in day^−1^) was estimated for each species, in each experimental microcosm:
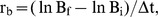
where B_f_ is the final population biomass (on day 30, in µg L^−1^), B_i_ is the initial population biomass (on day 0 or 10, in µg L^−1^, depending on the competition scenario), and Δt is the time interval (20 or 30 days).

### Statistical Analyses

The effect of salinity level on the competitive outcome of the experimental *Daphnia-Simocephalus* assemblage was only analyzed on the rate of population biomass increase (r_b_), because it is a more suitable estimate of competitive outcome [8]. Indeed, absolute abundance or biomass values on day 30 may merely reflect the fact that the one or the other species were introduced first (in scenarios S>D and D>S), while r_b_ expresses the rate at which they grew from the starting inoculum – making it comparable between species.

In order to assess which species grew better in each combination of competition scenario and NaCl concentration, we calculated the ratio between *Daphnia* and *Simocephalus* rates of increase for each microcosm. Subsequently, we assessed if these ratios significantly deviated from 1 (equal population growth) using independent one-sample *t*-tests. Because this required nine separate tests, we adjusted *p*-values so that they reflected the multiplicity correction [35], [36]; to do so, a two-stage procedure based on the control of false discovery rate [37] was employed, using the spreadsheet provided by Pike [36].

A two-way ANOVA on r_b_ data was used to analyse salinity and priority effects, using NaCl concentration and species inoculation order as fixed factors. These analyses were run separately for each species. Whenever an interaction between NaCl concentration and species inoculation order was found, a simple main effect analysis was carried out for species inoculation order, within each salinity level (using the error term of the two-way ANOVA as the denominator of the F-test; [35]). For this purpose, significance level was adjusted (α = 0.017) to control over the family-wise type I error rate, using the Dunn-Sidak procedure.

Except where noticed (see above), statistical analyses used a 0.05 significance level. Statistical software Minitab (v16) and SPSS (v17) were used.

## Results

Acceptable fluctuations in oxygen (range 8.5–10.8 mg L^−1^) and pH (range 7.6–8.5) levels were recorded in the microcosms, throughout the duration of the experiment. Within each NaCl level, conductivity was stable in the microcosms, ranging from 0.272 to 0.292 mS cm^−1^ (0.00 g NaCl L^−1^), 1.59 mS cm^−1^ (0.75 g NaCl L^−1^), and 2.84 to 2.86 mS cm^−1^ (1.50 g NaCl L^−1^). Salinity measurements did not vary at all: 0.0 (0.00 g NaCl L^−1^), 0.7 (0.75 g NaCl L^−1^) and 1.5 (1.50 g NaCl L^−1^).

**Figure 1:**
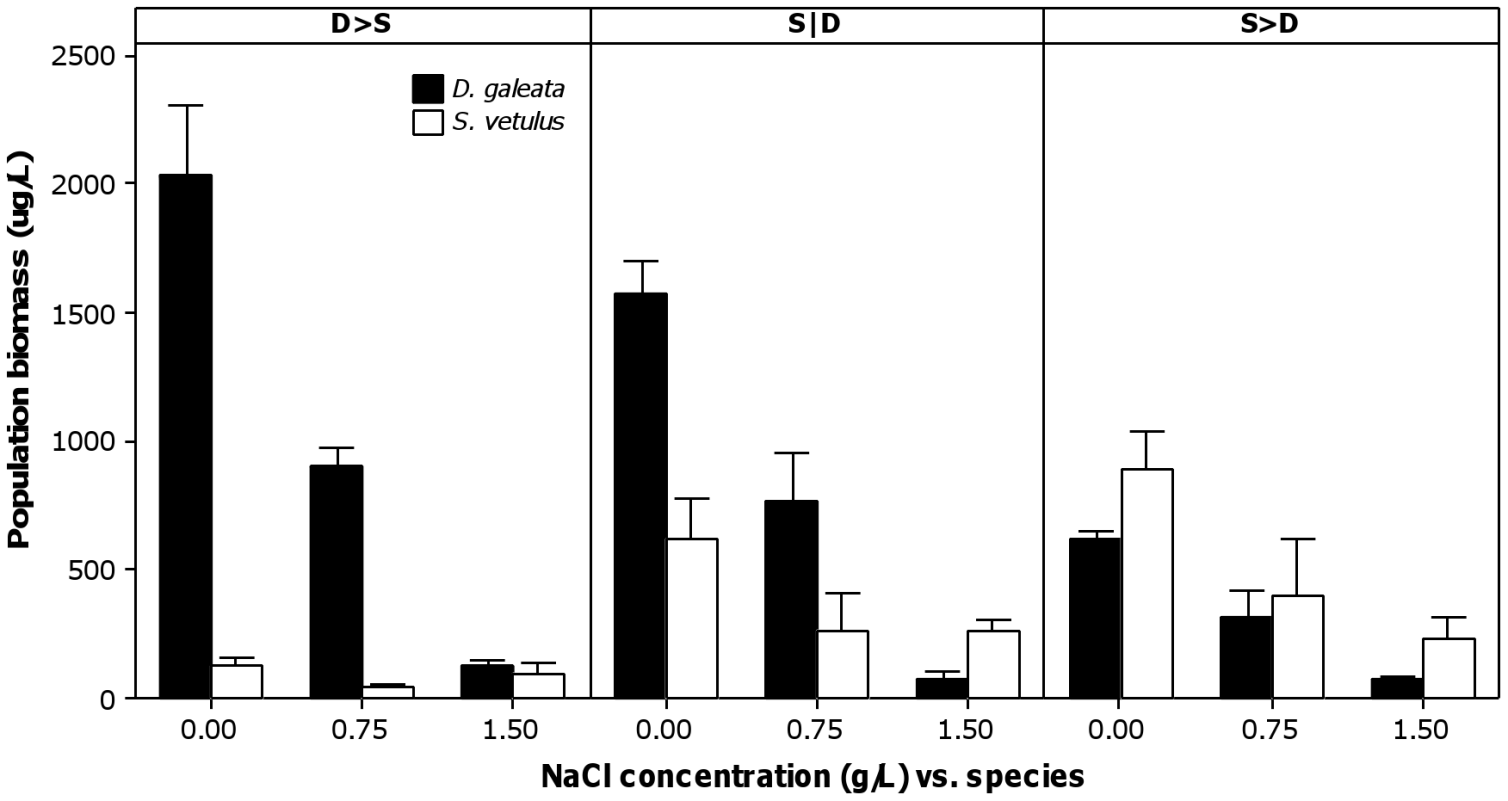
Average population biomass (in µg L ^−1^) at day 30 in different competition and salinity scenarios. Panels represent three competition scenarios, which differ in the order of inoculation of the species (see text for codes). Competitor species are shown as black (*Daphnia galeata*) and white (*Simocephalus vetulus*) bars. Error bars represent 95% confidence intervals of the mean (n = 3 experimental microcosms).

**Figure 2:**
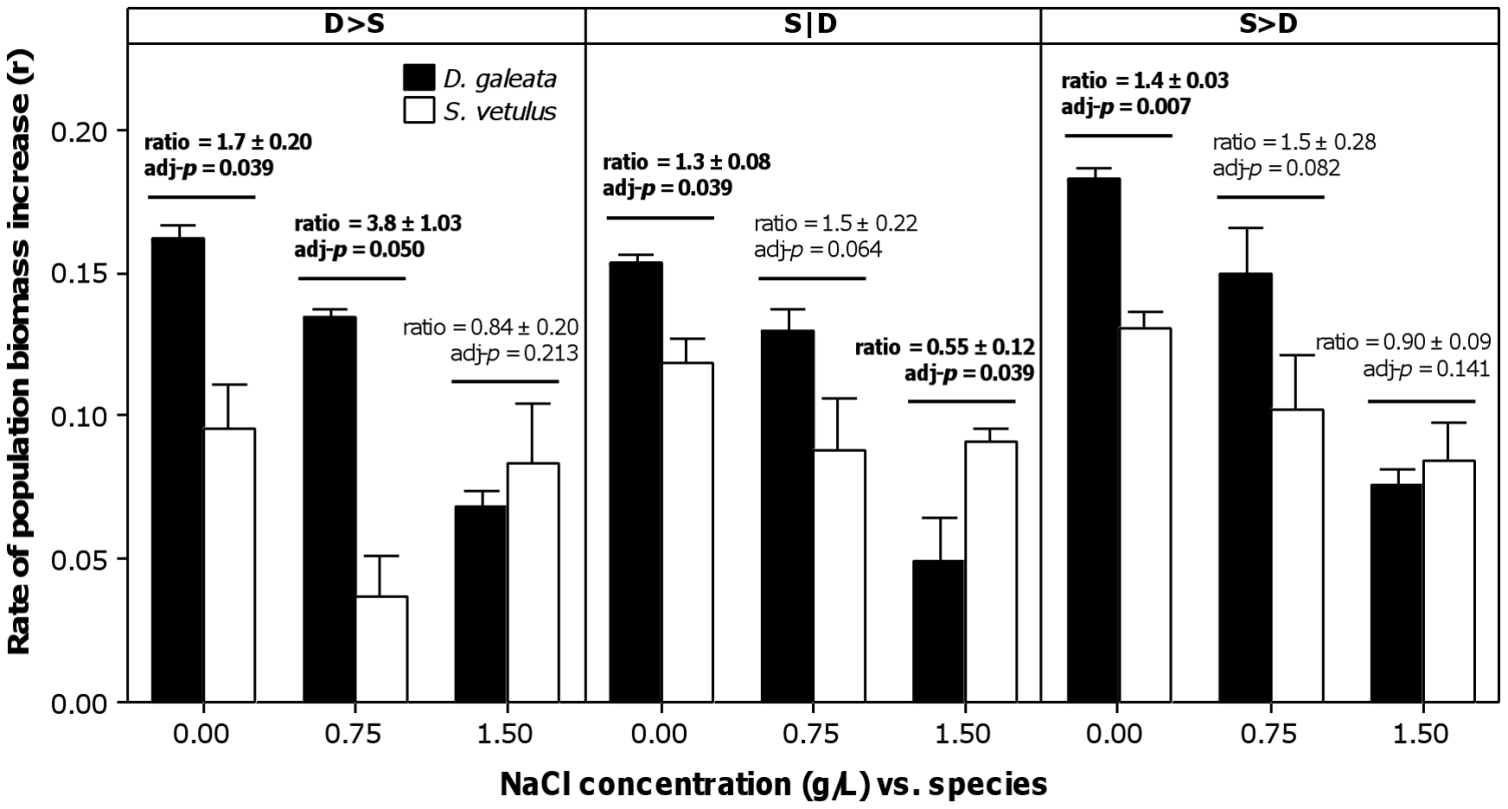
Average rate of population biomass increase (in day ^−1^) in different competition and salinity scenarios. Panels represent three competition scenarios, which differ in the order of inoculation of the species (see text for codes). Competitor species are shown as black (*Daphnia galeata*) and white (*Simocephalus vetulus*) bars. Error bars represent 95% confidence intervals of the mean (n = 3 experimental microcosms). Mean parwise ratios (and 95% confidence interval) of the biomass increase of competing species (*Daphnia:Simocephalus*) are shown, along with associated significance (adjusted for multiple comparisons, by controlling false discovery rate) for one-sample *t*-tests. Ratios that are significantly different from 1 are highlighted in bold: ratios significantly higher than 1 stand for higher biomass increase rate of *Daphnia* relatively to *Simocephalus*, whilst ratios <1 stand for the contrary scenario.

At the end of the experiment, different relative compositions of the experimental communities were obtained (Figure 1). *Daphnia* or *Simocephalus* were overall dominant in the experimental treatments where they were given chronological advantage in the inoculation order (respectively, D>S and S>D); this was mostly noticeable in the case of *Daphnia* for the D>S treatment. When both species were inoculated at the same time (S|D), *Daphnia* was generally the dominant taxon, suggesting it is competitively superior to *Simocephalus*. Salinity seemed to alter the relative composition of the communities, favouring *Simocephalus* in detriment of *Daphnia* (see data at 1.50 g L^−1^). To properly assess this, one must look at the rate of increase of the competing populations (see Statistical Analyses and Fig. 2).

At low salinity (0.00 g L^−1^), the ratios between *Daphnia* and *Simocephalus* rates of increase (r_b_) were significantly higher than 1, confirming *Daphnia* as a superior competitor (higher biomass increase rate – Figure 2), irrespective of the species inoculation order. This was also the case at 0.75 g L^−1^, in the competition scenario where *Daphnia* was inoculated first. At these salinity levels, priority effects thus seemed negligible, given the prevalence of *Daphnia* in all competition scenarios. However, at higher NaCl concentrations, this advantage was nullified or even inverted (in the case of S|D treatment at 1.50 g L^−1^). Apparently, *D. galeata* competitive abilities were compromised at the highest salinity level, with the competitive advantage being slightly on the side of *S. vetulus* under these conditions (Figure 2).

**Figure 3:**
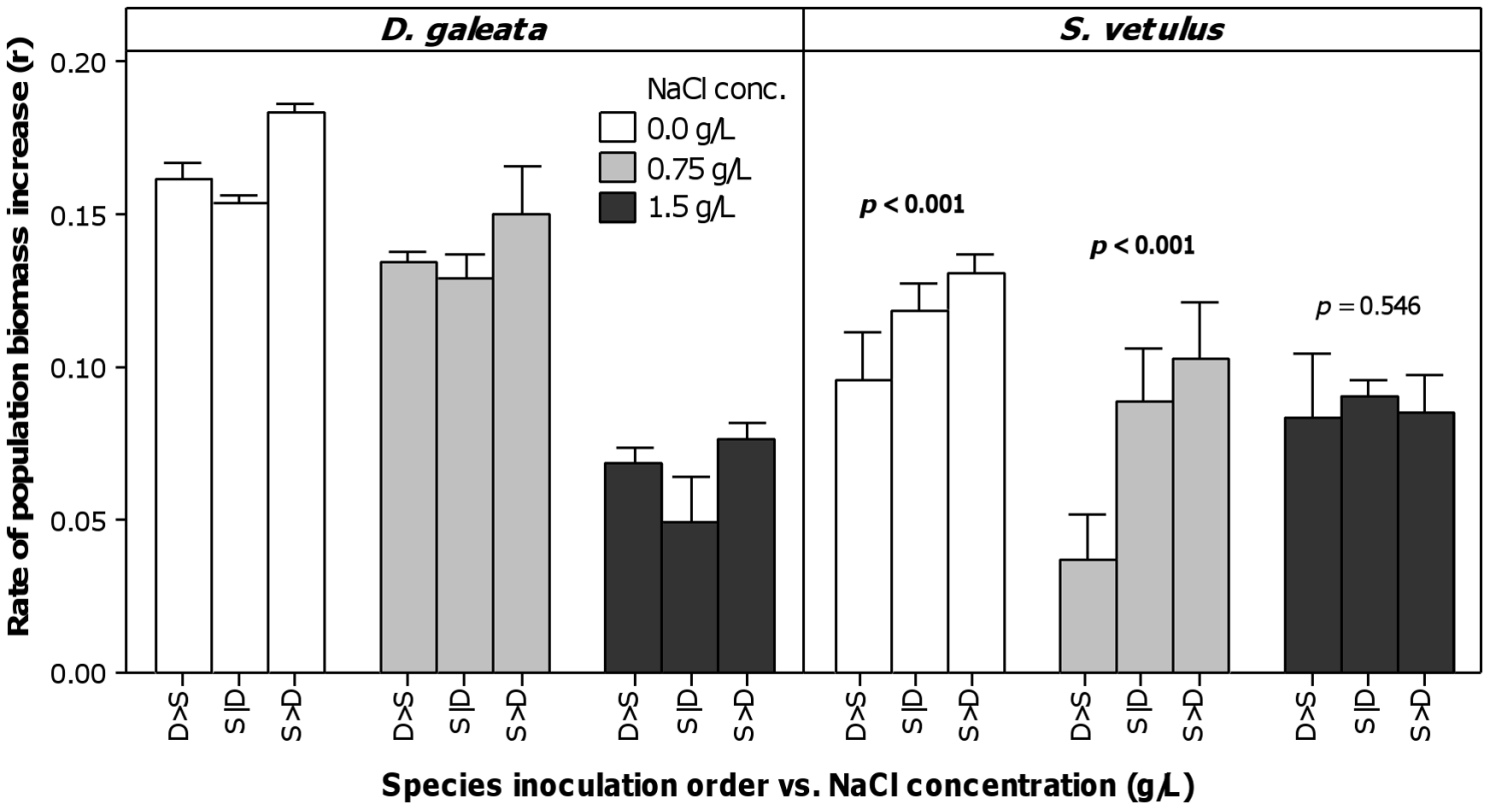
Average rate of population biomass increase (in day ^−1^) per competitor species. Groups of bars represent NaCl concentrations (in g L^−1^), which are crossed with competition scenario (species inoculation order, see text for codes). Error bars represent 95% confidence intervals of the mean (n = 3 experimental microcosms). For each salinity level, *p*-values for simple main effects of inoculation order are shown; significant values (α = 0.017, adjusted for multiple comparisons) are highlighted in bold.

Indeed, *Daphnia* biomass increase rate was mainly affected by NaCl concentration (Table 1 and Figure 3), decreasing monotonically from 0.00 to 1.50 g L^−1^. To a lesser extent (see *F*-ratios, Table 1), *Daphnia* increase rate was also affected by the inoculation order (i.e. competition scenario), with r_S>D_>r_D>S_>r_S|D_. Unlike for *Daphnia*, main effects of inoculation order (i.e. priority effects) were not consistent across salinity level for *Simocephalus*, as shown by the significant interaction between these two factors (Table 1). Both NaCl concentration and inoculation order were equally important as sources of variation of the *Simocephalus* biomass increase rate (see *F*-ratios). At 0.00 and 0.75 g L^−1^ NaCl, differences in *Simocephalus* population biomass increase rates were consistent (simple main effects, Figure 3), being maximum when priority was given to *Simocephalus* (S>D), intermediate when both species were inoculated at the same time (S|D), and minimum when *Daphnia* had the initial advantage (D>S). However, no significant differences were found among different inoculation orders at 1.5 g L^−1^ NaCl. At this NaCl level, *Simocephalus* population biomass increased at the same rate in all competition scenarios irrespective of the species inoculation order, suggesting that the competitive pressure imposed by *Daphnia* was alleviated.

**Table 1:** Summary of the two-way ANOVAs applied to the population biomass increase rate (*r*_b_) data, for each competitor species.

	*Daphnia*				*Simocephalus*			
Source of variation	df	MS	F	*P*	df	MS	F	*P*
NaCl concentration	2	0.024682	988.9	**<0.001**	2	0.003687	51.1	**<0.001**
Competition scenario	2	0.001488	56.9	**<0.001**	2	0.002921	40.5	**<0.001**
NaCl conc. × comp. scenario	4	0.000062	2.5	0.081	4	0.000833	11.5	**<0.001**
Residual	18	0.000025			18	0.000072		

Significant values are highlighted in bold.

## Discussion

This experimental study demonstrated a salinity-mediated disruption of the competitive outcome in *Daphnia*-*Simocephalus* microcosms. A shift between a *Daphnia*-dominated and a *Simocephalus*-dominated community occurred along the NaCl gradient. A similar result was found in experimental zooplankton communities when an invertebrate predator was introduced [8]. Subtle environmental changes, such as low levels of salinity, produce indirect effects in key ecological mechanisms, namely species sorting. Thus, our results support the hypothesized mechanism of differential species sorting within zooplankton communities in brackish environments or in freshwaters facing secondary salinization. Also, this study demonstrates that salinity, even at low levels, was much stronger than priority effects, which – in this case – were negligible because either *Daphnia* or *Simocephalus* were superior competitors, depending on the NaCl concentration.

Up to 0.75 g L^−1^, *Daphnia* demonstrated to be a superior competitor, independently of the order of inoculation (see Figures 2 and 3). Although the order of inoculation contributed to the overall variation in the rate of population biomass increase (see Table 1 and Figure 3), priority effects were negligible: *Daphnia* always grew best. This was also the case when *S. vetulus* competed with two other *Daphnia* species [8]. However, the order of inoculation was important for the inferior competitor, *Simocephalus*, whose populations grew worse when *Daphnia* was the early colonizer and grew best when it (*Simocephalus*) arrived earlier (see Figures 2 and 3). This demonstrates priority effects, with the competitive pressure on *Simocephalus* being higher when its competitor arrived earlier in the communities. Nevertheless, even at its highest growth rate, *Simocephalus* was never a match for *Daphnia* at low salinity (<1.5 g L^−1^). Therefore, we can consider that priority effects were negligible, as they did not translate into contrasting or long-lasting differences in species dominance. Although biomass data suggest such contrasting differences, this was merely a product of the short duration of the experiment, as shown by the rate of population biomass increase (compare Figs. 1 and 2), which is a more suitable estimate of competitive outcome (see Statistical analyses).

The superior competitor ability of *Daphnia* could be probably due to the successful establishment of its population through a rapid monopolization of resources [8], [38], ability to explore low levels of food [7], [39], [40], and superior filtration rate relatively to *Simocephalus* [8], [41]. Despite this, competitive exclusion [3], [4] of *Simocephalus* was not observed here in any scenario. However, the experiment was of relatively short duration and food levels were not very limiting (see [31]). Also, competition is not a force as radical as predation (see [8]), which implies that active removal of individuals from populations occurs. Priority effect in these two species in the field could occur due to earlier arrival of propagules (ephippia), which depend strongly on dispersal vectors (such as aquatic birds) [8], [9], [11]. While this is true for temporary ponds [8] (also in amphibians, e.g. [42]), it is not the case of lakes and reservoirs, which usually contain a large ephippial pool in the sediments [9]. In this case, priority effects could occur by differences in hatching time or in numbers (*Daphnia* typically produces two resting eggs per ephippium, while *Simocephalus* only produces one [38]).

While both populations’ growth rate decreased with increasing NaCl concentration, *D. galeata* growth was much more affected at 1.5 g L^−1^, and this alleviated *S. vetulus* from the pressure of a superior competitor. Consequently, priority effects were nullified, and *Simocephalus* experimental populations grew equally well in all species inoculation order scenarios at 1.5 g L^−1^. The superior competitive ability of *Simocephalus* at 1.5 g L^−1^ may have resulted from its higher chronic halotolerance relatively to *Daphnia*. Preliminary laboratorial tests (unpublished data) showed that the two taxa have similar acute EC_50_ values for NaCl –2.81 g L^−1^ (95% CI: 2.65–2.99 g L^−1^) for *S. vetulus* and 2.88 g L^−1^ (2.73–3.05 g L^−1^) for *D. galeata* – but the reproductive EC_50_ for the *S. vetulus* clone was slightly higher than the *D. galeata* clone used in the experiments –1.28 g L^−1^ (95% CI: 1.22–1.33 g L^−1^) and 0.71 g L^−1^ (0.64–0.77 g L^−1^), respectively. Thus, our results do not support the hypothesis that priority effects confer some protection to the less tolerant species (in this case, *Daphnia*). Similarly, a study with *Microcystis* populations in the presence of grazers also showed no protective effect of inoculation order in grazer-unprotected strains [12]. We conclude that the shift from a *Daphnia*-dominated (0.0 g L^−1^) to a *Simocephalus*-dominated assemblage (1.5 g L^−1^) was apparently mediated by their NaCl tolerance, resulting in depressed *Daphnia* growth at 1.50 g L^−1^ and consequent alleviation of competition pressure over *Simocephalus*, as seen by the lack of an effect of the inoculation order unlike in ≤0.75 g L^−1^ scenarios.

These evidences support the theory that the competition between species can be alleviated under environmental stress [16], favouring the inferior competitor or species, even if it arrives later to the community [6], [8], [12]. Consequently, as competitive strength is reduced, decreased impact of priority effects occurs in the presence of a stressor, such as predation [8], limiting food resources [6], or pesticides [42]. Similarly to our study, Louette and De Meester [8] showed that predation was responsible for an inversion of the dominant taxon in experimental communities. Although not as radical as predation (which lead to extinction of some species and hence negative growth rates in [8]), low salinity levels (1.5 g L^−1^) inverted the competitive outcome in the *Daphnia-Simocephalus* experimental system. The salinity levels at which this occurred are in line with the predictions for community shifts of Schallenberg et al. [23], as well as with the NaCl concentrations that elicit reproductive impairment in *Daphnia* [43], [44].

This study indicates that the higher halotolerance of certain genotypes/taxa could contribute to their success in disturbed communities, being important in the dynamics of species succession in a progressive scenario of freshwater salinization. In brackish lakes, large filter-feeding herbivores (especially *Daphnia* spp.) tend to be eliminated [23], [45]; consequently, smaller or more tolerant species dominate [6], [17] but their filtration efficiency is inferior, leading to lake regime shifts from clear to turbid water [25]. This rationale is applied here in the context of fish-populated lakes and reservoirs; it may not be true in brackish fishless ponds, where large-bodied *Daphnia* species that tolerate intermediate salinities occur (e.g. *D. magna*; see discussion in [44], [46]). Although *Simocephalus* is a large cladoceran, it is usually restricted to littoral environments and has a sessile behaviour [38]. Consequently, its filtration rate at whole-lake scale may not be efficient in controlling phytoplankton growth [8], [41]. Large cladoceran species, and particularly *Daphnia*, play a key role [25], [47] in the regulation of primary production in freshwater ecosystems (PEG model; [2]), because of their efficient algal filtration [39], [40], [47]. So, if the competitive ability of *Daphnia* species is compromised by external factors, such as demonstrated here for salinity, the dynamic of species succession could be modified, and the ecosystem services provided by these grazers (regulation of biogenic turbidity and prevention of cyanobacterial blooms, as well as nutrient cycling) would be nullified.

We must recognize that, in a scenario of moderate to intense salinization, the levels of salinity used in this study are not ecologically relevant. However, saline intrusion may elicit a progressive scenario, particularly in coastal lakes [23], [26], [48]. In these systems, small increases in salinity may occur due to intermittent inputs of seawater [23], [26], [49], but also via saline intrusion in groundwater, as the result of the conjugation of extended droughts [24], [50] and overexploitation of aquifers [24], [51]. Freshwater inland lakes can also suffer from salinization as result of extended drought and enhanced evaporation, especially in arid and semi-arid areas [46]. Thus, subtle or progressive changes in salinity may occur in freshwater systems, especially under a changing climate, and the potential impacts of small increases in salinity on biodiversity and trophic structure might be stronger than those of increased temperature per se [45]. This clearly justifies the need to assess the ecological consequences of such subtle changes in the resident assemblages, namely zooplankton, whose community structure is predicted to be highly sensitive to salinization (see [20], [21], [23], [49]).

Although our experiments used a simplistic experimental design, they demonstrated that gradual salinization of freshwater may alter competitive interactions in freshwater zooplankton, thus affecting the initial assemblage structure in colonization or re-colonization events. This occurs via interference with species sorting and priority effects. Other studies have also shown that the structure of communities reflects the environmental conditions in the moment of species sorting [6], [42], [52]; also, the environmental context is equally important in defining the community sensitivity to other stressors (e.g. pesticides) [53]. Future studies should therefore address the capacity of NaCl-altered zooplankton communities to cope with other stressors, as this could potentially compromise water quality (transparency, cyanobacterial blooms) and ecosystem functioning (e.g. primary productivity, nutrient cycling). Indeed, Wittebolle et al. [54] have shown that the initial assemblage structure is a key factor in preserving the resistance to environmental stress and functional stability of an ecosystem.
